# A cross-sectional study of job burnout, psychological attachment, and the career calling of Chinese doctors

**DOI:** 10.1186/s12913-020-4996-y

**Published:** 2020-03-12

**Authors:** Shu’e Zhang, Jinghui Wang, Fengzhe Xie, Dong Yin, Yu Shi, Min Zhang, Hongyan Yin, Fujun Li, Libin Yang, Depin Cao, Tao Sun

**Affiliations:** 1 Department of Health Management, School of Health Management, Harbin Medical University, Harbin, 150081 China; 2 Student Grade department, College of Bioinformatics Science and Technology, Harbin Medical University, Harbin, 150081 China; 3 Department of Health Management, School of Health Management, Harbin Medical University, Harbin, 150086 China; 4 Department of Anesthesiology, The first Affiliated Hospital, Harbin Medical University, Harbin, 150010 China; 5 Center for Higher Education Research and Teaching Quality Evaluation, Harbin Medical University, Harbin, 150081 China; 6 Department of Health Management, School of Medicine, Hangzhou Normal University, Hangzhou, 311121 China

**Keywords:** Chinese doctors, Job burnout, Psychological attachment, Career calling

## Abstract

**Background:**

The main objectives of this study were as follows: (1) evaluate the prevalence of burnout syndrome among doctors, (2) establish associations with demographic factors in China, and (3) examine the mediating role of psychological attachment in the relationship between job burnout and career calling.

**Methods:**

This cross-sectional survey was conducted by administering an online questionnaire in May 2016. The survey was performed across Thirty provinces. In total, A total of 3016 Chinese doctors were selected as participants, of which 2617 completed valid questionnaires (effective response rate: 86.77%).

**Results:**

The overall prevalence of burnout symptoms among Chinese doctors was 85.79%. Little variance was reported for burnout symptoms according to age (*Waldχ*^*2*^ = 6.843, *P* < 0.05, *OR* < 1), professional title (*Waldχ*^*2*^ = 13.110, *P* < 0.05, *OR* > 1), and daily working hours (*Waldχ*^*2*^ = 7.226, *P* < 0.05, *OR* > 1). However, the burnout of Chinese doctors was found to be associated with psychological attachment (*B* = − 0.6433, *P* < 0.0001) and career calling (*B* = 0.3653, *P* < 0.0001); furthermore, psychological attachment (*B* = 0.2350, *P* < 0.001) mediated the relationship between job burnout and career calling.

**Conclusion:**

Burnout symptoms among Chinese doctors were prevalent and associated with age, professional title, and long working hours. Chinese doctors aged 20–30 experienced a much higher level of burnout symptoms. The longer hours doctors worked, the more likely they were at risk of burnout symptoms, especially among attending physicians. Doctors who endured high-level burnout tended to exhibit decreasing psychological attachment, which threatened their sense of career calling. Finally, this paper proposed related explanations for the function mechanisms based on both theoretical and practical perspectives.

## Background

In recent years, a series of major changes related China’s medical system reform have taken place, especially regarding continuous improvement of medical service quality [1, 2]. However, considering China’s vast population and its increasing health claims, the country is facing a certain systemic crisis, including an insufficient number of doctors and an imbalanced structure of supply-demand within health services. Inescapably, Chinese doctors are suffering the stressful work conditions characterised by excessive workloads, high occupational stress, effort-reward imbalances, potential health risks, tolerance of customer’s rude behaviour, [3] and so on. Daily, they endure fatigue, frustration, and with patients while providing services [4], which increases burnout. To make matters worse, tired doctors can easily trigger further negative outcomes, [2, 5, 6] such as deterioration of doctor-patient relationships [7] and escalated violence in the health care sector [7]. Therefore, it is understandable that professional burnout among doctors has drawn continuous attention among academics and the public worldwide. Similarly, the various adverse effects resulting from doctor burnout also merit constant attention, which raises the question: given these deteriorating professional environments, how do doctors evaluate their own careers given that they are under tremendous stress and experiencing burnout? Particularly for doctors in highly challenging situations, ‘career calling’ can be regarded as a personal resource which provides their lives with meaning, increased resilience, and mechanisms for coping with challenges, further buffering the wider effects of burnout [8]. However, the never-ending series of patient complaints and array of negative emotions triggered by job burnout are likely to reduce doctors’ job satisfaction, organizational commitment, and psychological attachment to the hospital setting, ultimately resulting in lower career expectations and less motivation to pursue a career in medicine [8]. It is interesting to study how burnout affects doctors’ sense of career calling and whether psychological attachment plays a specific role in this relationship. However, the topic of the ‘career calling’ of doctors has received little international academic attention. This means that the more extensive adverse effects of doctor burnout are likely being ignored. Hence, the present study contributes to the literature by demonstrating the correlation between doctor burnout, psychological attachment, and career calling, as well as to verify possibly existing mechanism.

### What is ‘job burnout’?

‘Job burnout’ is regarded as a psychological syndrome involving chronic emotional and interpersonal stressors referring to individuals’ experiences at work and their subsequent responses to their tasks, organizations, co-workers, clients, and themselves [9, 10]. In the past half century, symptoms of burnout have been confirmed to exist within a wide range of human service professionals, and related research on job burnout has been undertaken extensively by scholars in various fields [9, 10]. A well-known and widely-accepted burnout model is Maslach’s three-dimensional model, [11] which includes variables such as emotional exhaustion, depersonalisation, and reduced personal accomplishment. Of these, emotional exhaustion is the core variable and is regarded as a kind of ‘negative’ mindset [11]. The Shirom-Melamed Burnout Measure is also popular among experts and serves as a well-known authority for explaining the issue of job burnout [12]. Pine believes that job burnout is an amalgam of both physical and psychological fatigue caused by long-term emotional deprivation [12]. Shirom and Melamed further define job burnout as a kind of mental state due to an exhaustion of physical, emotional, and cognitive energy [12]. Known as a ‘job killer’, burnout is likely to threaten an individual’s health, obstruct an organization’s performance, and may even cause serious social problems [13]. On the individual level, job burnout can cause serious problems such as headaches, insomnia, reduced work efficiency, impaired concentration, memory deterioration, poor immune system, [9] and so forth. Furthermore, mental health symptoms commonly caused by burnout have been verified and include anxiety, depression, and feelings of inferiority [9] which can lead to an employee’s poor quality of day-to-day working environment and family relationships as well as diminished self-image and happiness [13]. At an organizational level, an individual’s burnout symptoms are prone to influencing their team members, resulting in an overall deterioration of organizational morale and reduced efficiency of organizational operations [14]. A previous study has indicated that burnout may be linked to ‘escapism’ behaviour; for instance, job withdrawal and turnover may increase at significant cost to the organization [9]. Many studies have confirmed that the high prevalence of job burnout is closely related to worsening occupational environments, [9] which, in turn, causes a lack of harmony in interpersonal relationships and more occupational hazards [15]. However, the adverse effects caused by doctor burnout are a growing concern worldwide, and are far broader in scope than the results of this study demonstrate. Hence, further research still needs to be conducted, especially in healthcare setting where the effects of job burnout are potentially life-threatening [16].

### What happens when doctors experience job burnout?

The term ‘burnout’ has taken on a broad meaning that goes far beyond what is understood about it as a diagnosis or syndrome [17]. Therefore, how do we approach the issue of physician burnout in the medical profession? [18]. Burnout among doctors is an extensive problem, and their symptoms may not only threaten their health and sleep quality [10], but also the quality of the medical services that they provide and the well being of their patients, even their very lives, which restricts the advancement of China’s healthcare system reforms [15]. Numerous studies have found that doctor burnout is associated with a higher frequency of medical errors, lower patient satisfaction, longer post-discharge recovery times, and decrease in professional drive, lapses in professionalism, impeded learning, problematic alcohol use, and low job satisfaction [17]. Burnout tends to worsen and change over time, and the issue of doctor burnout is exhibiting an obvious increasing trend [17]. Today, there are still some problems in China’s health care system. To counterbalance what may be seen as dissatisfying reward for their efforts, some doctors are emphasising economic benefit rather than providing their patients with the best possible health care [19]. Even worse, there is a widespread sense that the career calling of doctors is gradually weakening, and the formerly trusting relationships between doctors and patients have been broken, which will be difficult to mend in the short term [1, 20]. An increase in doctors’ unprofessional and unethical behaviour induced by burnout could likely lead to more occurrences of violence towards doctors, [21] which also poses additional difficulties to reforming the healthcare system. The present study continues to focus on the current situation regarding burnout among physicians and its adverse results in the Chinese context in order to make a significant contribution to understanding the phenomenon of doctor burnout.

### What is the relationship between career calling, psychological attachment, and doctor burnout?

Career calling is defined as an approach to one’s work in which one is inspired by an ‘external summons’ and provided with a means through which one is able to derive meaning and fulfil positive social functions [8]. Specifically, career calling is related to so-called ‘career spirit [8]; that is, providing services for people, promoting physical and mental harmony, and improving people’s quality of life [8]. Vital research has found that career calling is linked to work effort, dedication, and well-being [22]. A sense of career calling is particularly important for doctors in highly challenging situations. Consequently, a correct and profound understanding of the medical profession is a necessary component of a career calling for any doctor [10, 23]. While there has already been much scholarly discussion about career calling, [23] these studies have mostly focused on understanding its core concepts [24]. There has been little research into the association between the career calling and burnout of doctors [10]. In China, where these issues are particularly problematic, questions of falling career calling and increased burnout are grossly neglected by academics and not reflected in systemic health care reforms. There is no doubt that it is essential to monitor these issues as they unfold and explore the relationship between doctors’ career calling and burnout in depth.

Doctors exhibit reduced enthusiasm for their work due to emotional exhaustion, a decline in professional meaningfulness, job satisfaction, and organizational identification [25]. When doctors are treated unfairly, their enthusiasm diminishes with in-role weakening and extra-role decreasing, [26] resulting a loss of psychological attachment to their organization [26]. *‘That special attachmen*t’, as Hirschman calls the loyalty psychologists term ‘effective commitment [27]—is an ‘employee’s emotional attachment to, identification with, and involvement in the organization [28, 29]. Doctors with high levels of attachment are prone to identifying with their career goals and values as well as a sense of pride regarding their profession; thus, they tend to undertake behaviour which extend beyond the job’s in-role requirements [30]. Therefore, we can assume that, if the degree of a doctor’s psychological attachment decreases, his or her acceptance of and emotional dependence on his or her sense of career calling (i.e., the reason he or she became a doctor in the first place) would diminish. However, previous studies have revealed that attachment plays a mediating role in many relationships [31]. Only a few studies have explored the notion that psychological attachment may mediate the relationship between job burnout and career calling among doctors. To remedy this gap in the literature, in this study, we examined psychological attachment using a function mechanism to test the mediating effect of psychological attachment. More importantly, the findings of this study contribute to new evidence increasingly revealing the adverse effects caused by the job burnout of Chinese doctors, specifically, negative effects on psychological attachment and career calling.

### What are the objectives of this study?

This study comprehensively investigated the following research questions: (1) how prevalent is burnout syndrome among doctors, and what differences in demographic factors can be established? (2) What is the relationship between job burnout, psychological attachment, and career calling? (3) How does job burnout threaten doctors’ career calling and does psychological attachment play a specific role in the relationship between burnout and career calling?

## Methods

### Subjects and procedures

A snowball sampling methodology was used to collect the data through the network investigation. Doctors from across China completed an anonymous online questionnaire in May 2016. A total of 3016 participants distributed across 30 cities participated in the survey, the HeiLongJiang (38.00%) province where the initial sample was recruited. The proportion of the other 29 provinces was ShanDong (17.00%), HuNan (6.00%), BeiJing (3.00%), GuangDong (3.00%), ZheJiang (3.00%), JiangSu (3.00%), LiaoNing (2.00%), JiLin (2.00%) and others (19.0%), and 2617 valid questionnaires were used as the samples (an effective response rate of 86.77%). First, a sampling frame was set up, approximately 50 doctors from the authors’ unit were selected as the original deliverers of the survey. Subsequently, they were fully informed of the purpose and significance of this study and their positive cooperation was obtained. Then the colleagues or classmates of ‘the original deliverers’ were invited to participate in our online survey. Meanwhile, the remaining doctors were colleagues invited by the initial participants. An anonymous online questionnaire was used to collect data, and anyone could obtain the Website Lucky Money as an award after they answered the questionnaire.

Our final sample was selected with strict adherence to exclusion criteria for data management and quality control. Inclusion criteria were as follows: doctors must be Chinese, give informed consent for participation, and participate voluntarily. Exclusion criteria included: no answer,voluntarily withdrawing from the study by failing to answer all the questions, too many missing items or obvious response errors, taking too little or long to respond.

### Representativeness and comparability

The general characteristics of the study participants (age, gender, service years, education level and professional title) were compared to those of Chinese doctors which were included in the Chinese Health and Family Planning Statistical Yearbook [32](CHFPSY) report on practicing physician published in 2016. Currently, this is the authority source concerning the characteristics of practicing physicians available. In addition, the results of the study participants group and the results of the CHFPSY members group were analyzed separately and then compared to determine the homogeneity of the sample [33].

### Measures

This study consisted of four questionnaires. Demographic variables were gathered using a self-designed questionnaire and included age, gender, education level, professional title, marital status, hospital level, service years, and so on.

### Measurement of job burnout

Li et al.’s Job Burnout Inventory with 15 revised items was used to measure the three dimensions of burnout (emotional exhaustion, depersonalization, and reduced personal accomplishment) [34]. This Job Burnout Inventory in medica settings was demonstrated to have good reliability and validity [35, 36]. Items were scored on a seven-point Likert scale ranging from 0 (‘totally disagree’) to 6 (‘totally agree’) where higher scores represented a higher degree of job burnout. The Cronbach’s alpha coefficient of the scale was 0.882. Maslach’s three-dimensional model includes (1) emotional exhaustion (EE), (2) depersonalization (DA), and (3) reduced personal accomplishment (PA) [11]. In this study, the scores of the three dimensions of burnout were as follows: (1) EE: low (< 11), medium (11–15), and high (> 15); (2) DA: low (< 9), medium (9–12), and high (> 12); and (3) PA: low (< 19), medium (19–22), and high (> 22) [35, 36]. Maslach’s cut-off scores for EE, DA, and reduced PA are 25, 11, and 16, respectively [37]. According to the scores for these three dimensions, the model then divides job burnout into four levels: (1) no burnout (all three scores are less than cut-off), (2) mild burnout (one of the three scores is greater than or equal to cut-off), (3) moderate burnout (two of the three scores are greater than or equal to cut-off), and (4) severe burnout (all three scores are greater than or equal to cut-off) [37].

### Measurement of psychological attachment

The psychological attachment inventory by Burris et al. with 4 items revised was used to measure the Chinese doctors’ psychological attachment level [31]. The cross-cultural adaptability of the psychological attachment inventory has been examined in other studies [38]. The psychological attachment inventory was applied to Chinese doctors for the first time. Items were scored on a five-point Likert scale ranging from 1 (‘totally disagree’) to 5 (‘totally agree’), where higher scores represented a higher degree of the psychological attachment. The Cronbach’s alpha coefficient of the scale was 0.920.

### Measurement of career calling

To assess doctors’ career calling level, we used the 4-item Career Calling Scale revised by Dik et al. [39]. The cross-cultural adaptability of the Career Calling Scale has been examined in other studie s in China [40]. The Career Calling Scale was applied to Chinese doctors for the first time. Items were scored on a five-point Likert scale ranging from 1 (‘totally disagree’) to 5 (‘totally agree’), where higher scores represented a higher degree of career calling. The Cronbach’s alpha coefficient of the scale was 0.786.

### Statistical analysis

All data were collected using a cross-sectional survey and analyses were performed using the SPSS 22.0 program (SPSS, Inc., Chicago, IL). Descriptive statistical analysis was used to describe the data collected. An internal consistency reliability test was performed to check inventory reliability, and logistic regression analysis was conducted to examine differences among demographic variables. We provided values including F, R2, and R2-changes, and the fit of the model was assessed with R2. Unstandardized regression coefficients (B), standard error (SE), and *P* values were reported for each step in the regression model. Statistical significance was defined as P < 0.05 (two-tailed).

### Ethics statement

The Institutional Review Board (IRB) of Harbin Medical University approved this study. Since the survey was anonymous, it was impossible to obtain informed written consent. In this case, an informed consent form was included at the beginning of the questionnaire. Completing the questionnaire was therefore considered ‘informed consent’ for participation in the survey. Confidentiality was maintained for all information collected in the survey.

## Results

### Demographic information for samples

The representativeness of the responding physicians was evaluated by comparing characteristics of the study participants with those of Chinese doctors which were published in Chinese Health and Family Planning Statistical Yearbooks, regarding age groups distribution (22.40% under 30 years in study participants vs 22.10% in the CHFPSY report), gender (47.40% vs 55.90% males), service years (16.50%under 30 service years in study participants vs 23.40% in the CHFPSY report) and education level (44.30% vs 51.70% bachelor in the CHFPSY report). In this study, participants’ demographic variables are shown in Table 1.

**Table 1:** Socio-demographic characteristics of the respondents (*n* = 2617)

Characteristic	Number	Percent
Age		
20–30	587	22.4
31–40	1224	46.8
41–50	658	25.1
51+	119	4.5
Unsure	29	1.1
Service Years		
0–10	1088	41.6
11–20	720	27.5
20+	376	14.4
Unsure	433	16.5
Hospital level		
Tertiary hospitals	1740	66.6
Second-class hospital	733	28.1
Primary hospital	139	5.3
Missing value	5	2.0
Gender		
Male	1240	47.4
Female	1369	52.3
Unsure	8	3.0
Education level		
College degree or below	291	11.1
Bachelor	1350	51.7
Master	692	26.5
Doctor	277	10.6
Unsure	7	3.0
Marital status		
Unmarried	397	15.2
Married	2148	82.1
Divorce or loss of spouse	70	2.7
Unsure	2	1.0
Professional title		
Without professional title	306	11.7
Resident doctor	564	21.6
Attending physician	898	34.4
Associate chief physician	569	21.8
Chief Physician	270	10.4
Unsure	10	4.0
Shift work		
Often work during the day	503	19.2
Occasional work at night	500	19.1
Often work at night	1613	61.6
Unsure	1	0.1
Daily working hours (hours)		
0–8	528	20.2
9–10	1297	49.6
11–12	504	19.3
13+	285	10.9
Unsure	3	0.1

### The incidence of job burnout and the three dimensions

The status of Chinese doctors’ job burnout is shown in Table 2. Among 2617 participating Chinese doctors, a total of 2245 reported experiencing varying degrees of job burnout over the past years. The overall prevalence of all degrees of burnout was 85.79%, and the breakdown according to severity is as follows: 713 (40.0%) mild, 1233 (27.2%) moderate and 299 (7.4%) severe burnout.

**Table 2:** Values are numbers (percentages) of respondents regarding job burnout (*n* = 2617)

Variables		N	%	M	SD
Emotional exhaustion	mild	193	7.4	19.35	6.24
	moderate	518	19.8		
	severe	1899	72.8		
Depersonalization	mild	726	27.8	12.89	5.50
	moderate	647	24.8		
	severe	1241	47.5		
Reduced personal accomplishment	mild	950	36.5	20.67	7.29
	moderate	329	12.6		
	severe	1327	50.9		
Job burnout	never	353	13.49	52.90	14.02
	mild	713	27.24		
	moderate	1233	47.12		
	severe	299	11.43		

### Total burnout-univariate analysis

The analysis of the factors influencing Chinese doctor job burnout is shown in Table 3. One of the classification variables was intentionally set as a ‘dummy’ variable. Univariate logistic regression analysis of the development dataset (2617 doctors) included in the equation was as follows: (1) job burnout as dependent variable (never = 0, exist = 1); (2) doctor’s age (20–30 = 1, 31–40 = 2, 41–50 = 3, 51+ = 4); (3) gender (male = 1, female = 2); (4) education level (college degree or below = 1, bachelors = 2, masters = 3, doctorate = 4); (5) professional title (without professional title = 1, resident physician = 2, attending physician = 3, associate chief physician = 4, chief physician = 5); (6) marital status (unmarried = 1, married = 2, divorced or loss of spouse = 3); (7) hospital level (tertiary hospitals = 1, secondary hospital = 2, primary hospital = 3); and (8) number of daily working hours (in hours). According to our results, most doctors (85.79%) exhibited varying degrees of job burnout. Age (Waldχ^2^ = 7.231, *P* < 0.01, OR < 1), education level (Waldχ^2^ = 9.751, *P* < 0.05, OR > 1), professional title (Waldχ^2^ = 14.969, P < 0.01, OR > 1) and daily working hours (Waldχ^2^ = 11.942, P < 0.01, OR > 1) were the factors most related to job burnout. These four factors were then entered into the multivariate logistic regression model.

**Table 3:** Univariate logistic regression analysis of respondents (*n* = 2617)

Variables	B	S.E.	Wald	Df	P	OR
Age (contrast = 20–30)						
31–40	0.158	0.151	1.101	1	0.294	1.172
41–50	−0.181	0.163	1.239	1	0.266	0.835
51+	−0.651	0.251	6.742	1	0.009	0.522
Gender (contrast = male)						
Gender: female	0.184	0.115	2.541	1	0.111	1.202
Education level (contrast = college degree or below)			9.751	3	0.021	
Education level: bachelor	0.303	0.172	3.101	1	0.078	1.354
Education level: master	0.605	0.197	9.450	1	0.002	1.832
Education level: doctor	0.288	0.231	1.553	1	0.213	1.333
Professional title (contrast = without professional title)			14.969	4	0.005	
Professional title: resident doctor	0.422	0.199	4.488	1	0.034	1.525
Professional title: attending physician	0.383	0.182	4.453	1	0.035	1.467
Professional title: associate chief physician	0.474	0.200	5.609	1	0.018	1.606
Professional title: chief physician	−0.132	0.216	0.371	1	0.542	0.877
Martial status (contrast = unmarried)			5.958	2	0.051	
Martial status: married	−0.370	0.176	4.411	1	0.036	0.691
Martial status: divorce or loss of spouse	0.211	0.458	0.213	1	0.644	1.235
Hospital level (contrast = tertiary hospitals)			2.993	2	0.224	
Hospital level: second-class hospital	−0.175	0.127	1.914	1	0.167	0.839
Hospital level: primary hospital	−0.302	0.239	1.588	1	0.208	0.740
Daily working hours	0.234	0.068	11.942	1	0.001	1.264

### Total burnout-multivariate analysis

Further screening of the factors influencing doctor job burnout are shown in Table 4. We analysed the four factors selected by the univariate logistic regression analysis using multivariate factor logistic regression analysis. According to Table 4, the result of the significance test of the whole regression model was *X*^*2*^ = 37.872 (*P* = 0.001*, P* < 0.01), while the result of the *Hosmer-Lemeshow* test was 2.132 (*P* = 0.977, *P* > 0.05); therefore, these regression models were optimal. As shown in Table 4, the results of the tests concerning the variables of age (*Waldχ*^*2*^ = 6.790, *P* < 0.05, *OR* < 1), daily working hours (*Waldχ*^*2*^ = 7.226, *P* < 0.01, *OR* > 1) and professional title (*Waldχ*^*2*^ = 13.110, *P* < 0.01, *OR* < 1) were significant, suggesting that these factors are the significant predictors of job burnout among Chinese doctors.

**Table 4:** Multivariate logistic regression analysis of respondents (*n* = 2617)

Variables	B	S.E.	Wald	Df	P	OR	Correlation strength
Daily working hours	0.187	0.070	7.241	1	0.007	1.206	Cox-Snell R2 = 0.015 Nagelkerke R2 = 0.027
Age (contrast = 20–30)			6.790	3	0.079		
31–40	−0.094	0.192	0.238	1	0.626	0.910	
41–50	−0.436	0.246	3.126	1	0.077	0.647	
51+	−0.742	0.329	5.087	1	0.024	0.476	
Education level (contrast = college degree or below)			3.332	3	0.343		
Education level: bachelor	0.132	0.192	0.477	1	0.490	1.141	
Education level: master	0.338	0.219	2.382	1	0.123	1.401	
Education level: doctor	0.044	0.263	0.028	1	0.867	1.045	
Professional title (contrast = without professional title)			9.287	4	0.054		
Professional title: resident doctor	0.329	0.213	2.392	1	0.122	1.390	
Professional title: attending physician	0.412	0.235	3.072	1	0.080	1.510	
Professional title: associate chief physician	0.696	0.280	6.191	1	0.013	2.005	
Professional title: chief physician	0.237	0.318	0.556	1	0.456	1.267	
Constant	1.123	0.243	21.288	1	0.001	3.073	
Overall model fit test	X2 = 37.805 Hosmer-Lemeshow =6.213						

### Psychological attachment as a mediator of the associations between job burnout and career calling

The means, standard deviations, and Pearson’s correlation coefficients of the continuous variables are presented in Table 5. All the variables were significantly correlated with one another, and job burnout was negatively correlated with psychological attachment (*r* = − 0.577, *p* < 0.01) and career calling (*r* = − 0.422, *p* < 0.01). There was also a positive correlation between psychological attachment and career calling (*r* = 0.543, *p* < 0.01). The absolute value of the correlation coefficient was between 0.25 and 0.65, which indicated that each variable could be used in the subsequent regression analyses.

**Table 5:** Means, standard deviations (SD), and correlations of continuous variables (*n* = 2617)

Variables	M	S D	Job burnout	Psychological attachment	Career calling
Job burnout	3.526	0.934	1.000		
Psychological attachment	2.698	1.134	−0.557^**^	1.000	
Career calling	3.020	0.959	−0.422^**^	0.543^**^	1.000

**P < 0.01, Correlation is significant at the 0.01 level (2-tailed)

Psychological attachment was tested as a possible mediator of the relationship between job burnout and career calling. The mediator was tested by calculating bias-corrected 95% confidence intervals using bootstrapping with *N* = 5000 resamples via the PROCESS procedure for SPSS22.0 [41]. Hierarchical linear regression analysis was performed to examine the relationship between job burnout, career calling, and psychological attachment (Table 6 M1–4) after eliminating the effects of demographic variables (age, gender, marital status, hospital level, professional title, educational level, and hospital department). This study regarded ‘job burnout’ as the independent variable, ‘psychological attachment’ as the mediator variable, and ‘career calling’ as the dependent variable. We found that psychological attachment had a significantly positive influence on career calling (B = 0.3653, *P* < 0.0001), and that the job burnout of Chinese doctors had a significantly negative influence on psychological attachment (B = − 0.6433, P < 0.0001) and career calling (B = − 0.3873, P < 0.0001). Moreover, psychological attachment played a mediating role between job burnout and career calling (B = − 0.3653, P < 0.0001). The indirect effect of job burnout on career calling was found to be − 0.2350, and the direct effect of job burnout on career calling through psychological attachment was found to be − 0.1523. The total effect of job burnout on career calling was − 0.3873.

**Table 6:** Hierarchical linear regression models of mediation (*n* = 2617)

Model	*B*	*SE*	*Test statistic*	*P-value*	*95% CI (LLUI-ULCI)*	
M1 Job burnout for Psychological attachment	−0.6433	0.0222	−28.9340	0.0001	*(−0.6869–0.5997)*	*R = 0.5570; R-sq = 0.3102;F = 1249.3620; p = 0.0001*
M2 Psychological attachment for career calling	0.3653	0.1830	19.9716	0.0001	(0.3296 0.4012)	R = 0.5504; R-sq = 0.3029; F = 114.4493; p = 0.0001
M3 Job burnout for career calling (direct effect)	−0.1523	0.0221	−6.9013	0.0001	(−0.1956–0.1090)	
M4 Job burnout for career calling (total effect)	−0.3873	0.0204	−19.0255	0.0001	(−0.4273–0.3474)	R = 0.4135; R-sq = 0.1710; F = 62.0979; p = 0.0001

The indirect effect of job burnout on career calling was − 0.2350

The direct effect of job burnout on career calling was − 0.1523

The total effect of job burnout on career calling was − 0.3873

## Discussion

### The status of the Chinese doctor job burnout

The results of the present survey indicated that the overall prevalence of burnout among Chinese doctors was 85.79% with a breakdown in severity as follows: 713 (40.0%) mild, 1233 (27.2%) moderate, and 299 (7.4%) severe. This result implied that Chinese doctors experienced different degrees of job burnout in the past year. Compared with the data from the United States [42] and Canada, [43] the symptoms of job burnout were the most serious among Chinese doctors. Additionally, compared with other professions, [44] Chinese doctors seemed to experience more prevalent burnout symptoms. Consequently, these results provided clear evidence that hospital managers as well as the Chinese government should pay more attention to this severe public health issue in the future.

### Influencing factors of Chinese doctor job burnout

The present study found that the level of job burnout among Chinese doctors was most significant for the variables of age, daily working hours and professional title. In China, doctors’ professional titles are developed by health administrations and hospitals to better manage physicians. They represent the medical skill level of doctors, which is generally divided into undetermined physician assistants, resident doctors, attending physicians, associate chief physicians and chief physicians from low to high. Younger doctors and attending physicians reported they had experienced more serious symptoms of job burnout, which is consistent with previous research [45]. When younger and attending physicians surpassed their physiological and psychological limits, they likely failed to meet excessive role demands [46]. During their first five years of medical training, young physicians are required to master complex procedural tasks and skills [10] as well as produce academic research. Stress, therefore, has an unquestioned significance in the burnout phenomenon, particularly when it is persistent [47]. In China, younger physicians, especially attending physicians, often suffer from all kinds of pressure in the workplace, such as role overload, unruly patients, and less work experience, which further results in job burnout [48]. In contrast, [42] older doctors with extensive experience who occupy higher positions are likely to receive more respect and adequate rewards as well as experience fewer role conflicts [42]. On the other hand, older doctors have mastered how to adjust their work rhythms and relieve stress, minimising the risk of job burnout [49]. In this sense, physicians are more at risk of experiencing burnout symptoms earlier in their careers [45].

One result of this study which was consistent with a previous study [45] was that the rates of severe job burnout were higher as working hours increased. On average, Chinese doctors work over 10 h per day [50]. In situations where the pressure to work longer hours is high, doctors have less time for sleep, personal relationships, and family activities [10]. Therefore, doctors may omit many duties and obligations from their family roles, which can contribute to higher stress and fatigue, greater work-life imbalance, and more work-family conflicts, [48] further aggravating burnout [51]. Moreover, doctors consume a large amount of physical and mental energy during their long hours of overtime, which facilitates the development of negative attitudes toward their work [52] and increases physiological fatigue and emotional exhaustion [46, 50, 53].

### Psychological attachment as mediators of the associations between job burnout and career calling

The results of the present study demonstrated the significant negative impact of job burnout on career calling, which is in accord with previous studies [10]. In addition, this study also found that psychological attachment played a partial mediating role between job burnout and career calling. A high level of job burnout decreased doctors’ psychological attachment toward their organization and reduced their career calling.

According to the stress theory, [54] doctors’ stressful professional contexts largely foster a kind of professional burnout which can be considered as part of an individual’s process of changing his or her professional attitudes and behaviours in a negative manner [55]. In China, the many problems in hospital settings include poor working conditions, work overload, lack of appropriate remuneration, and a malfunctioning health care system,which may help explain why many excellent younger doctors develop negative professional attitudes and behaviours as well as to low-psychological attachment [47]. Psychological attachment can invigorate the doctors’ spirits because it encourages doctors to make efforts on behalf of their medical organization. However, as seen in this study, when experiencing a high level of burnout, doctors reduced their professional investment, and did not take on extra efforts for their organization, and developed a negative attitude towards their work. If doctors’ psychological attachment to their organization decreased due to burnout, it is not surprising, that a sense of a ‘calling’ was no longer an occupational goal,so the doctors no longer viewed their careers as a calling.

As the Conservation of Resources (COR) Theory pointed out, when doctors lost their resources or invested them but did not reap the expected reward, they exhibited psychological pressure and detachment from their organization [56]. Burnout appears when doctors lose their resources and are unable to supplement their cognitive, emotional, and physical abilities [47]. The high level of job burnout among doctors provides a relatively low assessment of gains leisure and vital, spiritual, familial, material, and political resources, which can lead to further detachment from their medical service [56]. As detachment occurs, problems such as physicians being ‘physically uninvolved in services, cognitively unvigilant, and emotionally disconnected from others in ways that hide what they think and feel, their creativity, and beliefs and values’ likely arise. Therefore, the act of ‘providing services for people’ is no longer regarded as part of the doctor’s career goals and motivations [10]. Even worse, it can further affect doctors’ beliefs, attitudes, and behaviours about their careers with possible consequences for career identity and dependence as well as the norms and spirit of Chinese health care overall. Increased job burnout subsequently reduces the degree of a doctor’s psychological attachment to his or her organization, increasing professional turnover and also lowering the sense of medicine as a ‘calling’. Therefore, hospital managers should pay greater attention to symptoms of job burnout in subordinate doctors, and make significant efforts toward developing strategies that strengthen doctors’ psychological attachments to their organization as well as their sense of career calling.

### Limitations

This study offered a number of interesting discoveries. However, some limitations should be considered. Firstly, this cross-sectional survey was conducted by administering with an online questionnaire, and a snowball sampling methodology was used, which increased the potential for sampling bias, e.g., there are differences in the response rate of doctors in different provinces. Secondly, the cross-sectional nature of this study prevented establishment of causation related to the causal relationship between job burnout, psychological attachment, and career calling; ultimately, these results could not be regarded as describing a causal relationship. Thirdly, data were collected from the self-reports of doctors, which introduced response bias from social desirability or negative affection. Finally, we used foreign scales that ignored issues of cross-cultural adaptability, which offers scope for academic attention in the future. The issue of job burnout is common in hospital settings, and its influence is extensive and pervasive; therefore, longitudinal studies should be conducted in the future.

## Conclusions

This study revealed the prevalence of burnout syndrome among doctors (85.79%) in China’s hospital settings and its related factors. A key to implementation of our study’s findings is that hospital managers should take effective measures to prevent and relieve the symptoms of doctor burnout, especially focusing on doctors who are prone to job burnout, such as younger physicians and attending physicians with longer daily working hours.

According to our survey, Chinese doctors encountered burnout frequently, leading to negative effects such as a low degree of psychological attachment to their organization and sense of medicine as a ‘career calling’. In addition, this study was found that job burnout threatened career calling by weakening doctors’ psychological attachment to their organization. Another key to implementation of our study’s findings is to enhance career calling of doctors by strengthening doctors’ attachment to medical organizations. Namely, it is high time to take some measures to give doctors more humanistic care, improve their career development path and reduce their daily working hours in order to enhance their job attachment and career calling. Definitively, some new perspectives for future research were also provided by this study.

## Data Availability

Data is currently not available online, but the data can be obtained upon request from hydzhangshue@163.com.

## Abbreviations

B: Unstandardized regression coefficients
CHFPSY: Chinese Health and Family Planning Statistical Yearbook
COR: Conservation of Resources
DA: Depersonalization
EE: Emotional Exhaustion
M: Mean
PA: Personal Accomplishment
SD: Standard Deviations
SE: Standard Error
